# P-247. Skills in Managing Vascular Catheters: Differences Between Self-Perception and Assessment by an External Auditor

**DOI:** 10.1093/ofid/ofae631.451

**Published:** 2025-01-29

**Authors:** Valeria Paes Lima, Regiane Cardoso, Sheila Silva, Ana Cláudia Fernandes, Rafael Andrade, André Augusto Ramos, Marcelo Ramos, Cristiane da Silva, Rodrigo Garbero

**Affiliations:** Hospital Sirio Libanês Brasilia, Brasília, Distrito Federal, Brazil; Hospital Sirio Libanês Brasília, Brasília, Distrito Federal, Brazil; Hospital Sírio Libanês Brasília, Brasília, Distrito Federal, Brazil; Hospital Sírio Libanês Brasília, Brasília, Distrito Federal, Brazil; Hospital Sírio Libanês Brasília, Brasília, Distrito Federal, Brazil; Hospital Sírio Libanês Brasília, Brasília, Distrito Federal, Brazil; Hospital Sírio Libanês Brasília, Brasília, Distrito Federal, Brazil; Hospital Sírio Libanês Brasília, Brasília, Distrito Federal, Brazil; Hospital Sírio Libanês Brasília, Brasília, Distrito Federal, Brazil

## Abstract

**Background:**

Implementing prevention strategies can be challenging for health services, and it is useful to know new assessment and teaching methods so that educational interventions are more effective.

Adherence to the intravenous medication administration checklist through self-assessment

**Figure:**
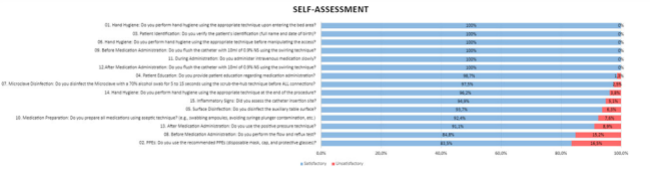

**Methods:**

Seventy-eight nursing professionals from a inpatient unit were invited to carry out an assessment of skills in intravenous medication administration. Initially, professionals completed a self-assessment questionnaire, in which they were asked to accurately report what corresponds to their usual practice. Then, the same professional administered the medication in a simulated environment while being observed by a trained professional, who filled out a standardized checklist. The observers were clinical nurse educator, an infection control nurse and a vascular access reference nurse. At the end of the procedure, the observer provided individual feedback to the professional on the positive aspects and non-conformities. Then, the professionals answered a questionnaire to evaluate this training modality.

Adherence to the intravenous medication administration checklist by an external observer

**Figure:**
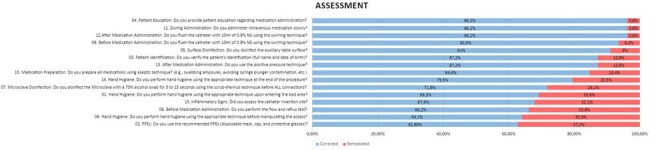

**Results:**

In the self-assessment questionnaire, the professionals attributed unsatisfactory practice to 4%, increasing to 19% in the evaluation of the process by an external observer. The average time to respond to the self-assessment questionnaire was 8 minutes and 48 seconds, and to observe the procedure was 11 minutes and 26 seconds. The results are shown in figures 1 and 2.

The difference in the assessment of hand hygiene drew attention: professionals attributed it to be 99% adequate, and external observers, 70.9%. The main non-conformities observed were the failure to apply hand hygiene in the 5 recommended moments, the use of incorrect or incomplete technique and the length of the nails or the presence of dirt. The assessment of catheter insertion was carried out by only 67.9% of professionals.

**Conclusion:**

The difference between self-perception and external assessment indicates the need for more innovative training methods with practical implementation, rather than the simple exposition of technical content. There was a good response from professionals to this method, and we consider the opportunity to provide individualized feedback in a safe environment very valuable.

**Disclosures:**

**All Authors**: No reported disclosures

